# Impact of human immunodeficiency virus type 1 ribonuclease H inhibitors on the polymerase and RNase H function of foamy virus reverse transcriptase

**DOI:** 10.1186/1742-4690-10-S1-P101

**Published:** 2013-09-19

**Authors:** Angela Corona, Anna Schneider, Kristian Schweimer, Paul Rösch, Enzo Tramontano, Birgitta M Wöhrl

**Affiliations:** 1 Department of Life and Environmental Sciences, University of Cagliari, Cittadella di Monserrato SS554, 09042, Monserrato (Cagliari), Italy; 2 Lehrstuhl Biopolymere, Universität Bayreuth, Bayreuth, Germany

## Background

In foamy viruses (FVs) only the integrase domain is cleaved off from the Pol precursor protein, resulting in a multifunctional mature reverse transcriptase (RT) harbouring the protease (PR) domain at its N-terminus. In contrast to the heterodimeric RT of human immunodeficiency virus type 1 (HIV-1), which consists of a 66 kDa and a 51 kDa subunit, the PR-RT of FVs is a monomeric protein. Thus, we wanted to analyse the inhibitory effect of HIV-1 nonnucleoside and RNase H inhibitors on the enzyme functions of FV PR-RT.

## Results

We tested the effect of five HIV-1 RNase H inhibitors, known to interact with different HIV-1 RT pockets [1,2], on the PR-RT of prototype FV (PFV). All compounds inhibited the PFV RNase H function at concentrations comparable to the ones reported for the HIV-1 RNase H, suggesting pocket similarity between the two proteins. In addition, four inhibitors also inhibited the PFV associated DNA polymerase activity. This can probably be attributed to differences in the protein’s structure and flexibility. The non-nucleoside RT inhibitor Efavirenz was inactive on PFV PR-RT, implying the absence of a binding pocket similar to the one in HIV-1 RT. NMR titration and *in silico* docking experiments with the free PFV RNase H domain and the diketo acid derivative RDS1463 identified a putative binding site reaching into the RNase H active site. Sequence alignments and structural overlays of HIV-1 and PFV RNase H were performed to identify the corresponding binding site in HIV-1 RNase H.

## Conclusions

We showed that HIV-1 RNase H inhibitors are able to inhibit PFV PR-RT RNase H activity and in addition polymerase functions. NMR titration and docking experiments suggest a putative binding site for the RNase H inhibitor RDS 1643 close to the PFV RNase H active site.
